# Six-minute walk test distance at time of hospital discharge is strongly and independently associated with all-cause mortality following cardiac surgery

**DOI:** 10.1038/s41598-024-52601-7

**Published:** 2024-01-30

**Authors:** Md Shajedur Rahman Shawon, Benjumin Hsu, Richard Chard, Ian A. Nicholson, Victoria L. Elias, Lauren K. Nicola, Corrina R. Moore, Andrew D. Hirschhorn, Louisa R. Jorm, Sean F. Mungovan

**Affiliations:** 1 Centre for Big Data Research in Health, University of New South Wales, Sydney, Australia; 2 Department of Cardiothoracic Surgery, Westmead Private Hospital, Westmead, NSW Australia; 3 Faculty of Medicine, University of Sydney, Sydney, NSW Australia; 4 Westmead Private Hospital Physiotherapy Services, Westmead Private Hospital Sydney, Westmead, NSW Australia; 5 MQ Health, Faculty of Medicine, Health and Human Sciences, Macquarie University, Maquarie Park, NSW Australia; 6 The Clinical Research Institute, Westmead, NSW Australia; 7 Department of Professions, Faculty of Health, Arts and Design, Swinburne University of Technology, Hawthorn, VIC Australia

**Keywords:** Cardiology, Prognosis

## Abstract

We investigated the impact of distance covered in the six-minute walk test (6mWT) before being discharged from the hospital after cardiac surgery on the risk of all-cause mortality. Our study included 1127 patients who underwent cardiac surgery and then took part in a standardised physiotherapist-supervised inpatient rehabilitation programme during 2007–2017. The percentage of the predicted 6mWT distance, and the lower limit of normal distance was calculated based on individual patients’ age, sex, and body mass index. We used Cox regression with adjustment for confounders to determine multivariable-adjusted hazard ratios (HRs) for mortality. Over a median follow-up period of 6.4 (IQR: 3.5–9.2) years, 15% (n = 169) patients died. We observed a strong and independent inverse association between 6mWT distance and mortality, with every 10 m increase in distance associated to a 4% reduction in mortality (HR: 0.96, 95% CI 0.94–0.98, P < 0.001). Those in the top tertile for predicted 6mWT performance had a 49% reduced risk of mortality (HR: 0.51, 95% CI 0.33–0.79) compared to those in the bottom tertile. Patients who met or exceeded the minimum normal 6mWT distance had 36% lower mortality risk (HR: 0.64, 95% CI 0.45–0.92) compared to those who did not meet this benchmark. Subgroup analysis showed that combined CABG and valve surgery patients walked less in the 6mWT compared to those undergoing isolated CABG or valve surgeries, with a significant association between 6mWT and mortality observed in the isolated procedure groups only. In conclusion, the longer the distance covered in the 6mWT before leaving the hospital, the lower the risk of mortality.

## Introduction

Physical functional capacity is an important clinical indicator for adults with cardiovascular disease^1,2^. A reduced physical functional capacity is associated with decreased quality of life and adverse clinical outcomes, including cardiac events, an increase in hospital admissions and all-cause mortality^3–6^. Increasing physical functional capacity is a part of the continuum of care and is important for patients who undergo cardiac surgery including coronary revascularisation and/or heart valve surgical procedures^2,7^. The direct measurement of peak oxygen consumption (VO_2_) during cardiopulmonary exercise testing (CPET) is regarded as the gold standard for physical functional capacity assessment^6,8^. However, the direct measurement of peak VO_2_ during CPET after cardiac surgery has low utility, is time- and resource-intensive, requires specialised equipment, and not routinely available in acute hospital and rehabilitation clinical settings. An alternative estimate of physical functional capacity in patients who undergo cardiac surgery is the six-minute walk test (6mWT).

The 6mWT is safe and practical, easy to perform, clinically accessible, and inexpensive^7^. The distance a patient can walk in six minutes is typically measured in a hospital corridor or hallway^9,10^. The 6mWT is well-tolerated by cardiac surgery patients in acute hospital and rehabilitation settings^8,11^. The distance walked during the 6mWT estimates the capacity of the patient to undertake independent functional mobility and activities of daily living^8^. In patients with heart failure^12–14^, chronic obstructive pulmonary disease^15,16^, pulmonary arterial hypertension^17^, and lung cancer^18^, the distance walked during the 6mWT is significantly associated with all-cause mortality. Early after cardiac surgery the estimated physical functional capacity of patients can be affected by perioperative factors, including prolonged bed rest, pain, anaemia, and restrictive respiratory patterns^19^. Currently it is not known whether the distance walked during the 6mWT early after cardiac surgery is associated with all-cause mortality. Therefore, the quantification of the relationship between the 6mWT distance and the risk of all-cause mortality for patients who have undergone cardiac surgery is clinically important and relevant. The distance walked during the 6mWT could be used to predict all-cause mortality in patients who undergo cardiac surgery and to identify high-risk cardiac surgery patients to optimise their post-acute hospital discharge care. Previous studies have reported conflicting results between the 6mWT distance and all-cause mortality in cardiac surgery patients undergoing cardiac rehabilitation^11,20–22^. These studies had limitations including, small sample sizes, limited or incomplete follow-up, and potential selection bias arising from the collection of all-cause mortality data.

Therefore the aims of this study were to: (i) determine the association between 6mWT distance at the time of acute care hospital discharge and all-cause mortality in cardiac surgery patients and (ii) determine if the association between the 6mWT distance at the time of acute hospital discharge and all-cause mortality varied according to sex and the type of cardiac surgery, including revascularisation, valve surgery or combined procedures. We hypothesised that patients who were able to walk further at the time of hospital discharge following cardiac surgery were less likely to die during long-term follow-up.

## Patients and methods

### Study population

This study was based on the Cardiothoracic Physiotherapy Database (CuPID) which prospectively collects data from all patients undergoing cardiac surgery at Westmead Private Hospital in New South Wales (NSW), Australia. The CuPID includes a cohort of patients who underwent cardiac surgeries between January 2007 and December 2017. All CuPID patients received an intensive physiotherapist-supervised phase 1 cardiac rehabilitation program delivered according to a standardised seven-day clinical pathway. The details of the standardised phase 1 cardiac rehabilitation program for CuPID patients have been reported elsewhere^23^. In brief, all patients participated in twice-daily sessions of physiotherapist-supervised physical activity during their entire postoperative acute hospital stay. The physical activities included respiratory techniques, active upper and lower limb musculoskeletal movements, and walking for up to 10 min per session, depending on each patient’s clinical status.

### Study design

The CuPID data were electronically linked to routinely collected administrative hospital and mortality data, providing unbiased follow-up on mortality for patients who have undergone cardiac surgery. The CuPID dataset was linked to the NSW Admitted Patient Data Collection (APDC) and NSW Registry of Births, Deaths and Marriages datasets. The APDC dataset contains information on admissions to all public and private hospitals in NSW, Australia. In this dataset, clinical diagnoses are coded according to the Australian modification of the International Statistical Classification of Diseases and Related Problems, 10th Revision (ICD-10-AM), and the procedures are coded according to the Australian Classification of Health Interventions (ACHI)^24^. Data linkage was performed probabilistically by the NSW Centre for Health Record Linkage (https://www.cherel.org.au/). De-identified records including a project-specific person number were provided to the researchers. The reported accuracy of data linkage by probabilistic matching exceeds 99%^25^.

**Figure 1:**
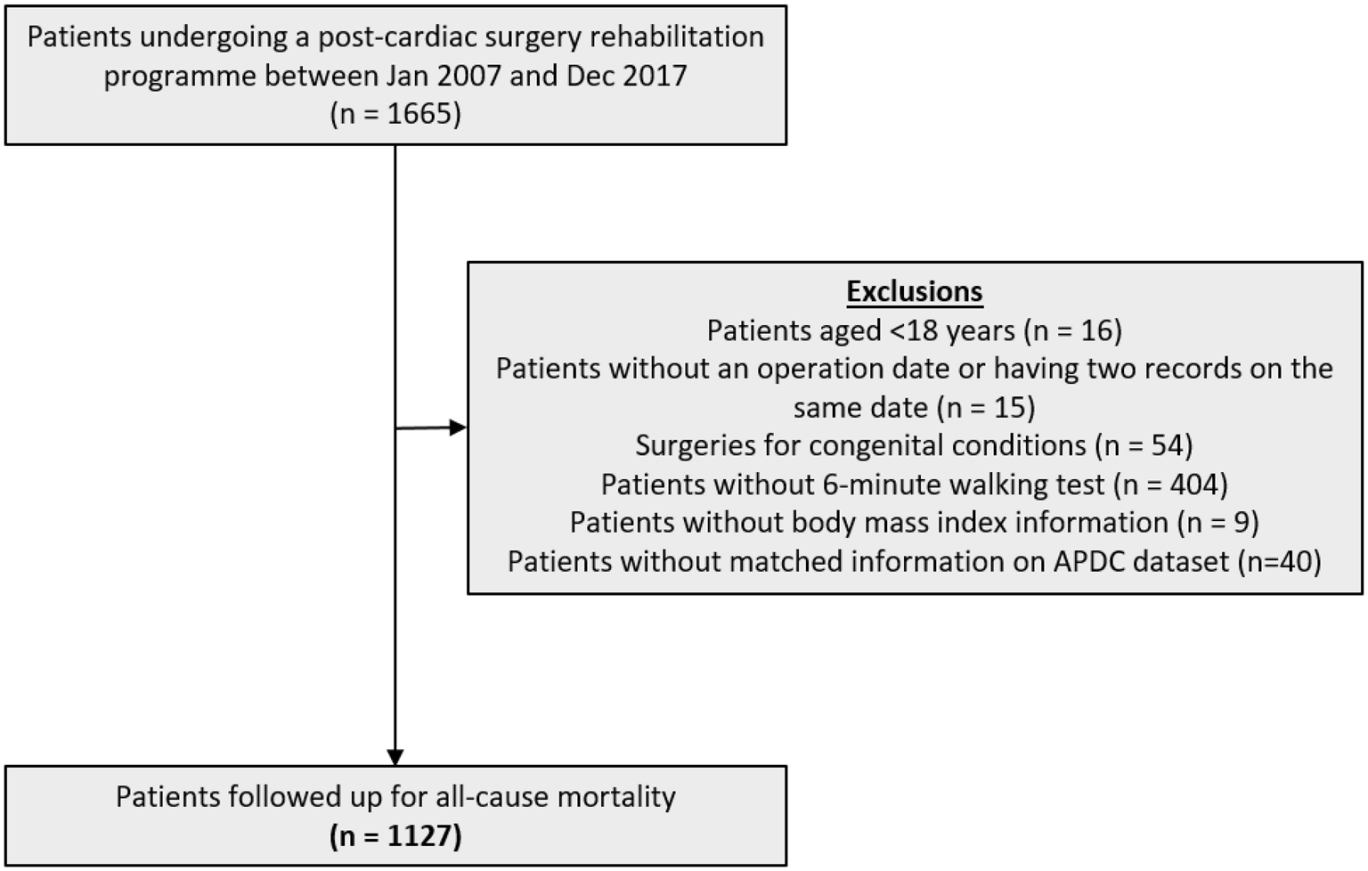
Flowchart showing cohort selection.

Patients were excluded if they were aged under 18 years, had surgeries for congenital heart conditions, did not have an operation date recorded or had two records with the same operation date, and did not complete the 6mWT before hospital discharge (Fig. 1). Nearly 400 records in the CuPID dataset did not have 6mWT-related information, indicating either that these patients did not perform pre-discharge 6mWT or that this information was not recorded. Patients who did not perform or have a pre-discharge 6mWT recorded were more likely to be older and female and to have urgent cardiac surgery procedures, longer postoperative lengths of stay, and more postoperative complications than those who had 6mWT information (Supplementary Material, Table S1). The operation dates in the CuPID and APDC datasets were exactly matched for 89% of the cohort. Among the patients without an exact date match, more than half had a discrepancy of ± 1 day between the records (Supplementary Material, Fig. S1). For the remaining patients, we cross-checked the ICD-10 codes in primary diagnosis field, patients’ insurance status, and hospital type before inclusion in the present study.

Our study was granted ethical approval by the University of New South Wales, NSW Population and Health Services Research (HREC/18/CIPHS/56), Aboriginal Health and Medical Research Council of NSW (1503/19), and Australian Institute of Health and Welfare (EO2018/2/431) research ethics committees. Each patient provided informed consent for the use of their data for research purposes during their inpatient phase 1 cardiac rehabilitation, and the research adhered to the principles set forth in the Declaration of Helsinki.

### Six-minute walk test

A single 6mWT was performed using a standardised protocol immediately following the phase 1 cardiac rehabilitation program and prior to hospital discharge. The patients were instructed to walk continuously at a self-selected pace in an enclosed airconditioned 43.5 m corridor for 6 min while maintaining a rating of perceived exertion of 3 (moderate) to 4 (somewhat strong) on the Borg CR-10 scale^26^. The supervising physiotherapist advised each patient of the elapsed time each minute. No other feedback or encouragement was provided during the test. However, patients were allowed to rest, if required. The total distance walked was measured to the nearest 0.5 m.

For analysis, we used the absolute distance walked in metres (m) and the percentage of the predicted distance walked calculated based on age, sex, and body mass index (BMI)^9^. The percentage-predicted 6mWT distances were divided into tertiles. We also estimated the lower limit of a normal 6mWT distance using reference equations accounting for age, sex, and BMI^9^ and then categorised patients according to whether they reached the lower limit of the normal distance or not.

### Study covariates and follow-up data

The CuPID includes information on patients’ height, weight, BMI, smoking status, slow vital capacity, and blood pressure measured before hospital discharge by the treating physiotherapist, as well as operative variables, postoperative length of stay and complications. Information about Elixhauser comorbidities^27^ (a set of 31 comorbidities) was collated from primary and additional diagnosis fields recorded in the index hospitalisation or any hospitalisation up to 12 months previously.

The primary outcome measure of our study was all-cause mortality. Mortality data were obtained from the NSW Registry of Births, Deaths and Marriages. The patients were followed from the day of completing the pre-discharge 6mWT to the earliest of the following: date of death or last follow-up date in the mortality data (31 December 2018). Person years were calculated based on the individual follow-up times.

### Statistical analysis

The patients’ descriptive characteristics were summarised as frequencies and percentages for categorical variables and as means and standard deviations (SD) or medians and interquartile ranges (IQR) for continuous variables.

Cox proportional hazard regression was used to estimate the hazard ratios (HR) with 95% confidence intervals (CIs) for the association between the 6mWT and all-cause mortality. The association between the 6mWT and all-cause mortality was examined using (i) absolute distance walked in meters as a continuous variable, (ii) tertiles of percentage-predicted 6mWT as a categorical variable, with tertile 1 (worst performers) used as the reference group, and (iii) completion status for the lower limit of the normal 6mWT distance as a binary variable (yes/no). An unadjusted model was first created, followed by an age- and sex-adjusted model and then by a fully adjusted model that included all sociodemographic, anthropometric, comorbidity, operative and postoperative variables. Kaplan–Meier survival curves were derived according to the tertiles of the percentage-predicted 6mWT distance and completion status for the lower limit of the normal distance. The association between the 6mWT and all-cause mortality was separately examined according to sex and types of cardiac surgery. The scaled Schoenfeld residuals were used to assess the proportional hazards assumption (Supplementary Material, Fig. S2).

All statistical analyses were performed using Stata version 16.0. Values of *P* < 0.05 were considered statistically significant. All *P*-values were two-tailed.

## Results

### Baseline patient characteristics

The CuPID dataset included 1665 patients undergoing cardiothoracic surgeries between 2007 and 2017. After all exclusions (Fig. 1), 1127 patients were included in the analysis. Among them, 53.3% of patients had undergone isolated coronary artery bypass graft (CABG) surgery, 32.8% had valve surgery and 9.7% had combined CABG and valve surgery. Urgent surgery was performed on 26.7% of patients, while 9.2% were re-intervention procedures. The patients were followed up for a median of 6.4 (IQR: 3.5–9.2) years, and 169 (15%) died during the follow-up period.

Selected baseline characteristics of the study population are shown in Table 1. The mean (SD) age at the time of surgery was 64.2 (13.1) years and a quarter of all patients were female. The mean (SD) BMI was 28.8 (5.1) kg/m^2^. Patients who died during the follow-up period were significantly older than those who survived (71.9 (SD: 9.6) vs. 62.8 (SD: 13.2) years). Compared those cardiac surgery patients who survived during the follow-up period, those cardiac surgery patients who died were more likely to be current or past smokers, have more Elixhauser comorbidities, significantly longer perfusion times (mean (SD) duration: 89.1 (40.4) vs 82.1 (37.4) min, p = 0.027), significantly greater postoperative hospital lengths of stay (mean (SD) duration: 9.9 (4.8) vs. 8.2 (2.8) days, p < 0.001) and a greater frequency of postoperative complications (Table 1).

**Table 1:** Selected baseline characteristics, overall and by outcome status.

	Overall	Survived during follow-up	Died during follow-up	P-value
Patient characteristics				
No. of patients (% of total)	1127 (100)	958 (85)	169 (15)	
Age (years), mean (SD)	64 (13)	63 (13)	72 (10)	< 0.001
Female, n (%)	285 (25)	243 (25)	42 (25)	0.887
Height (m), mean (SD)	1.7 (0.1)	1.7 (0.1)	1.7 (0.1)	0.059
Weight (kg), mean (SD)	83.3 (17.2)	83.7 (17.0)	81.2 (17.9)	0.080
BMI (kg/m^2^), mean (SD)	28.8 (5.1)	28.8 (5.1)	28.4 (5.3)	0.316
Current or past smoker, n (%)	459 (41)	370 (39)	89 (53)	< 0.001
Previous cardiac surgery, n (%)	347 (31)	292 (31)	55 (33)	0.592
Comorbidities				
Elixhauser comorbidities, n (%)				< 0.001
0–1	314 (28)	287 (30)	27 (16)	
2–4	711 (63)	596 (62)	115 (68)	
5 or more	102 (9)	75 (8)	27 (16)	
Operative variables				
Operation performed, n (%)				0.041
CABG	601 (53)	519 (54)	82 (49)	
CABG + valve	109 (10)	83 (9)	26 (15)	
Valve	370 (33)	314 (33)	56 (33)	
Others	47 (4)	42 (4)	5 (3)	
Urgent procedure, n (%)	301 (27)	254 (27)	47 (28)	0.725
Reintervention procedure, n (%)	104 (9)	91 (10)	13 (8)	0.454
Operation time (mins), mean (SD)	215 (71)	216 (69)	213 (86)	0.605
Perfusion time (mins), mean (SD)	83 (38)	82 (37)	89 (40)	0.027
Total ventilation time (hrs), mean (SD)	10.2 (14.4)	9.9 (14.6)	11.5 (13.5)	0.198
Postoperative variables				
Post-operative length of stay (days), mean (SD)	8.5 (3.2)	8.2 (2.8)	9.9 (4.8)	< 0.001
Postoperative complications, n (%)	266 (24)	213 (22)	53 (31)	0.010
Discharge variables				
Slow vital capacity (Litres), mean (SD)	2.2 (0.7)	2.2 (0.7)	2.1 (0.6)	0.056
SpO_2_, mean (SD)	96 (5)	96 (5)	96 (3)	0.765
Systolic blood pressure (mmHg), mean (SD)	126 (19)	126 (18)	126 (21)	0.759
Diastolic blood pressure (mmHg), mean (SD)	70 (10)	71 (10)	69 (12)	0.049
Mean arterial pressure (mmHg), mean (SD)	90 (13)	90 (12)	90 (15)	0.959
Home discharge, n (%)	922 (82)	800 (84)	122 (72)	< 0.001
Discharge 6mWT variables				
6mWT distance (m), mean (SD)	359 (99)	367 (97)	312 (100)	< 0.001
% of predicted 6mWT value (in tertiles)^a^, n (%)				0.431
Tertile 1	376 (33)	315 (33)	61 (36)	
Tertile 2	376 (33)	317 (33)	59 (35)	
Tertile 3	375 (33)	326 (34)	49 (29)	
Did not complete 6mWT lower limit of normal^a^, n (%)	562 (50)	481 (50)	81 (48)	0.585

^a^Estimated using equations reported by Enright et al.^9^.

*BMI* body mass index, *SpO*_*2*_ peripheral capillary oxygen saturation, *CABG* coronary artery bypass graft, *6mWT* six-minute walk test.

### Six-minute walk test performance

The patients walked a mean (SD) 6mWT distance of 359 (99) m at the time of hospital discharge. Half of all patients were unable to achieve the lower limit of the normal 6mWT distance estimated based on their age, sex, and BMI. An examination of baseline characteristics according to the tertiles of the percentage-predicted 6mWT showed that patients who covered the smallest 6mWT distances (tertile 1) were more likely to be older, female, have a lower BMI, undergo previous cardiac surgery, and have more Elixhauser comorbidities than patients in the other two tertiles (Supplementary Material, Table S2). The patients in tertile 1 were more likely to have re-intervention procedures, with longer average operation, perfusion, and ventilation times than patients in tertiles two and three.

### Six-minute walk test and all-cause mortality

**Figure 2:**
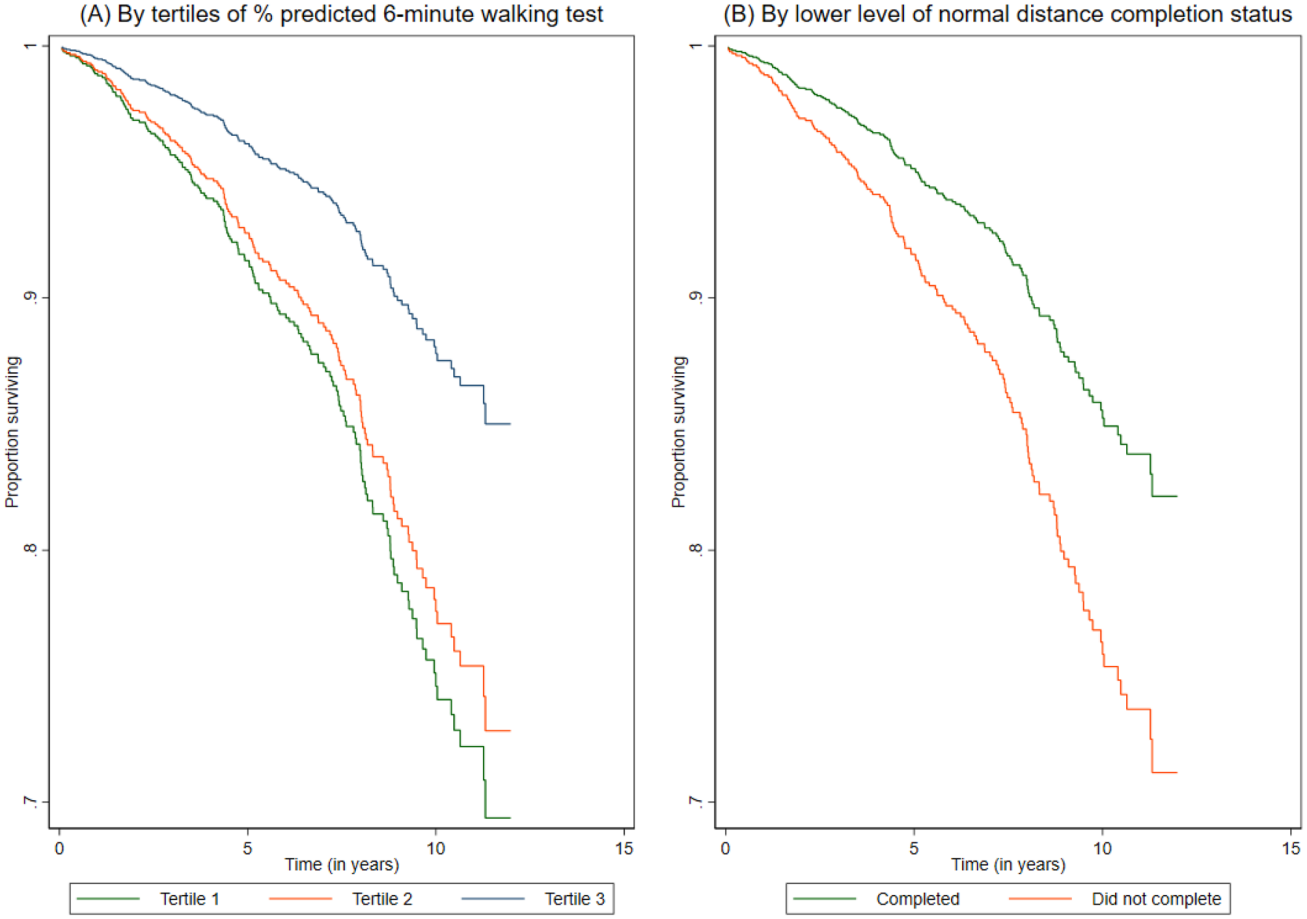
Age- and sex-adjusted survival curves for (**A**) tertiles of the percentage predicted 6-min walk test distance and (**B**) completion status of lower level of normal 6-min walk test distance.

**Figure 3:**
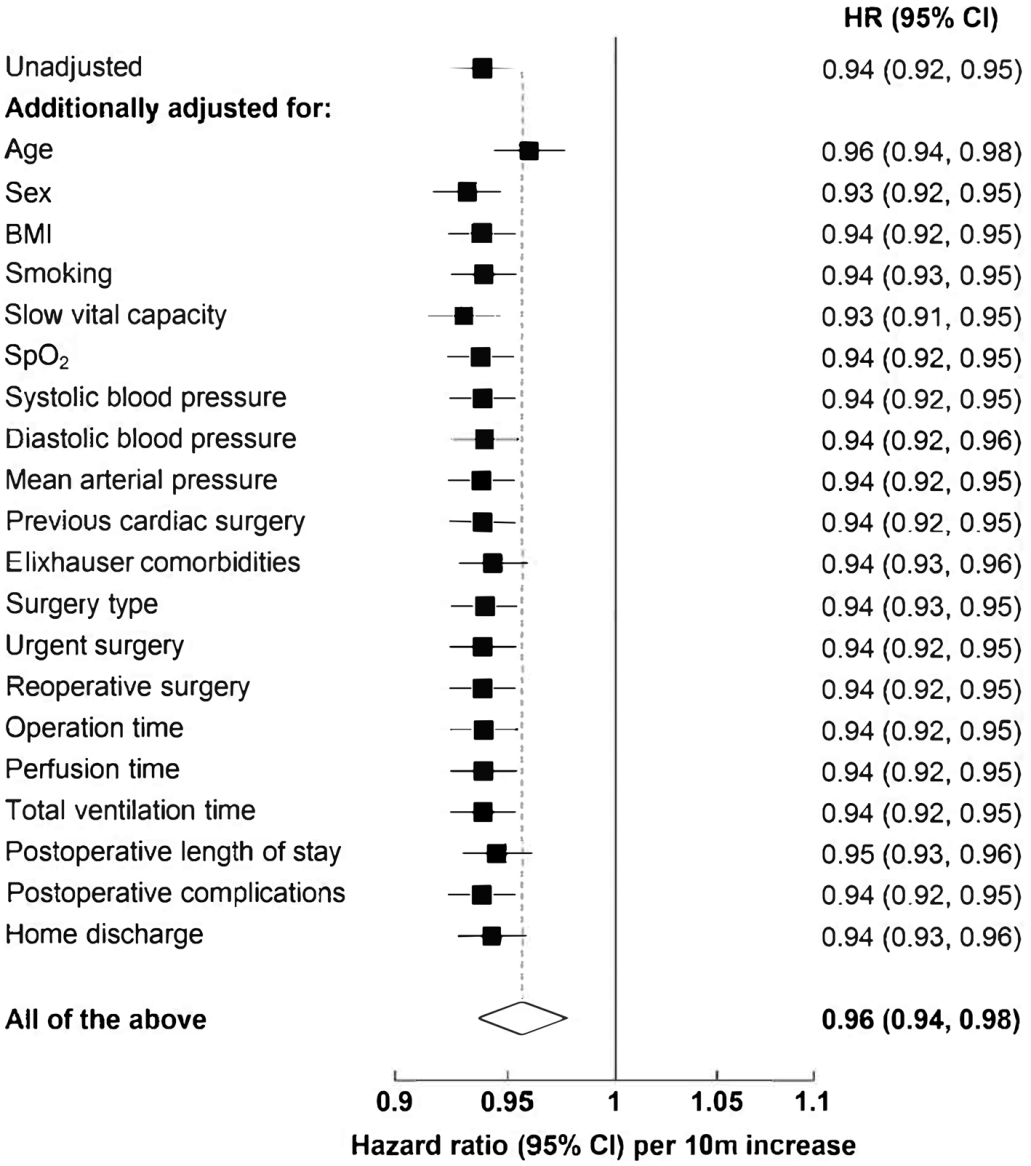
The effect of adjustment for covariates on the hazard ratio per 10 m increase in six-minute walk test for all-cause mortality.

Figure 2 presents the Kaplan–Meier survival curves according to 6mWT distances. Patients in tertile 3 (best performers) of the percentage-predicted 6mWT distance had a significantly lower hazard of dying over the follow-up period than patients in tertiles 1 (worst performers) and 2 (intermediary performers) (Fig. 2A). Patients who did not achieve the lower limit of the normal 6mWT distance were more likely to die than those patients who did achieve the lower limit 6mWT distance (Fig. 2B). Results from fully adjusted models showed an inverse relationship between the distance covered during the 6mWT and the hazard of mortality (multivariable-adjusted HR per 10 m distance increase: 0.96, 95% CI 0.94–0.98; Table 2). Compared to patients in tertile 1 of the percentage predicted 6mWT distance (worst performers), patients in tertile 3 (best performers) had a lower hazard of death (HR: 0.51, 95% CI 0.33–0.79) during the follow-up period. Patients who achieved the lower limit of the normal 6mWT distance also had a lower hazard of mortality than those who did not (HR: 0.64, 95% CI 0.45–0.92; Table 2). Separate analyses for male and female patients showed that the association was stronger among females than among males (HR per 10 m distance increase in the 6mWT: 0.89, 95% CI 0.85–0.94 vs. 0.97, 95% CI 0.95–1.00; Table 2). The association between the 6mWT distance and all-cause mortality remained strong when sociodemographic, anthropometric, comorbidity, operative and postoperative variables were added to the unadjusted model either individually or simultaneously (Fig. 3).

**Table 2:** Association between 6-min walking test and all-cause mortality.

Variables	Unadjusted model		Age and sex-adjusted model		Fully-adjusted model	
	HR (95% CI)	P-value	HR (95% CI)	P-value	HR (95% CI)	P-value
Total						
Per 10 m increase in 6mWT	0.94 (0.92–0.95)	< 0.001	0.95 (0.94–0.97)	< 0.001	0.96 (0.94–0.98)	0.000
% Predicted 6mWT						
Tertile 1	1.00 (Ref)		1.00 (Ref)		1.00 (Ref)	
Tertile 2	0.82 (0.56–1.20)	0.297	0.82 (0.56–1.21)	0.325	0.81 (0.54–1.22)	0.315
Tertile 3	0.57 (0.38–0.85)	0.005	0.45 (0.30–0.67)	0.000	0.51 (0.33–0.79)	0.003
Lower level of normal						
Completed	0.87 (0.63–1.20)	0.394	0.59 (0.42–0.81)	0.001	0.64 (0.45–0.92)	0.015
Did not complete	1.00 (Ref)		1.00 (Ref)		1.00 (Ref)	
Males						
Per 10 m increase in 6mWT	0.94 (0.92–0.95)	< 0.001	0.96 (0.94–0.99)	< 0.001	0.97 (0.95–1.00)	0.020
% Predicted 6mWT						
Tertile 1	1.00 (Ref)		1.00 (Ref)		1.00 (Ref)	
Tertile 2	0.94 (0.60–1.46)	0.773	1.03 (0.66–1.61)	0.907	1.03 (0.64–1.66)	0.912
Tertile 3	0.62 (0.39–0.99)	0.045	0.55 (0.34–0.88)	0.013	0.64 (0.38–1.08)	0.098
Lower level of normal						
Completed	0.87 (0.60–1.26)	0.460	0.62 (0.43–0.91)	0.013	0.68 (0.45–1.03)	0.068
Did not complete	1.00 (Ref)		1.00 (Ref)		1.00 (Ref)	
Females						
Per 10 m increase in 6mWT	0.92 (0.89–0.95)	< 0.001	0.93 (0.89–0.96)	< 0.001	0.89 (0.85–0.94)	0.000
% Predicted 6mWT						
Tertile 1	1.00 (Ref)		1.00 (Ref)		1.00 (Ref)	
Tertile 2	0.48 (0.20–1.13)	0.094	0.46 (0.19–1.08)	0.076	0.31 (0.11–0.86)	0.024
Tertile 3	0.44 (0.20–0.97)	0.041	0.30 (0.13–0.68)	0.004	0.20 (0.07–0.55)	0.002
Lower level of normal						
Completed	0.80 (0.41–1.56)	0.509	0.51 (0.25–1.03)	0.061	0.46 (0.20–1.07)	0.072
Did not complete	1.00 (Ref)		1.00 (Ref)		1.00 (Ref)	

Cox proportional hazard models were used to estimate hazard ratios (HRs) with 95% confidence intervals (CIs).

*HR* hazard ratio, *CI* confidence interval, *6mWT* six-minute walk test.

The subgroup analysis by surgery type showed that patients who had combined CABG and valve procedures walked significantly shorter distances during the 6mWT (316 m) than those patients who had isolated CABG (366 m) or valve procedures (358 m; p < 0.01; Table 3). Although the HRs per 10 m increase in 6mWT distance were similar for all three types of cardiac surgery, the association between 6mWT distance and all-cause mortality reached statistical significance in isolated CABG (HR: 0.94, 95% CI 0.91–0.98) and valve only surgery patients (HR: 0.94, 95% CI 0.91–0.98), but not in combined CABG-valve surgery patients (HR: 0.94, 95% CI 0.88–1.01) (Table 3).

**Table 3:** Association between 6-min walking test (6mWT) and all-cause mortality, by surgery type.

	Isolated CABG	CABG + valve	Valve only
6mWT (metres), mean (SD)	366 (94)	316 (93)	358 (103)
HR (95% CI) per 10 m increase in 6mWT distance	0.94 (0.91–0.98)	0.94 (0.88–1.01)	0.94 (0.91–0.98)

Cox proportional hazards models were used to estimate hazard ratios (HRs) with 95% confidence intervals (CIs).

*6mWT*  6-min walk test.

## Discussion

Our study investigated the association between the 6mWT distance at time of acute care hospital discharge and all-cause mortality in a contemporary cohort of patients undergoing cardiac surgeries between 2007 and 2017. We found that the 6mWT distance, expressed as an absolute value in metres and as a percentage of the predicted value, was inversely and significantly associated with the hazard of all-cause mortality. Patients who achieved the lower limit of the predicted 6mWT distance, based on age, sex, and BMI had a significantly lower hazard of death. The inverse association between the 6mWT distance and all-cause mortality was stronger in females than males. The findings from our study supports the use of the 6mWT for (i) the assessment of physical functional capacity and (ii) discharge planning prior to acute care hospital discharge following cardiac surgery.

Physical functional capacity, estimated by the 6mWT distance is associated with all-cause mortality in heart failure patient cohorts^12–14^, however the association following cardiac surgery is not well reported. In our study, we found that for every 10 m increase in 6mWT distance, there was a 4% decrease in the hazard of all-cause mortality over a median follow-up period of 6.4 years. Patients who completed the lower limit of normal 6mWT distance had a lower hazard of all-cause mortality (36%) compared to those who failed to complete the estimated 6mWT distance. We also found that a greater 6mWT distance was more strongly associated with a lower hazard of all-cause mortality among females than among males. Two previous studies^11,21^ have reported the predictive value of the 6mWT on mortality in a patient cohort receiving cardiac rehabilitation after cardiac surgery. La Rovere et al.^21^ found that a 1% increase in the predicted value of the 6mWT performance was associated with a 3% reduction in the risk of all-cause mortality over a median follow-up period of 23 months. Cacciatore et al*.*^11^ reported that completing a distance of 300 m or more during the 6mWT predicted a lower risk of mortality among patients aged ≥ 65 years (HR: 0.34, 95% CI 0.10–0.79, p = 0.033) but not among those aged < 65 years (HR: 0.76, 95% CI 0.31–2.12, p = 0.654). The results from these two studies^11,21^ are however not directly comparable to the present study due to differences in study design, patient selection, follow-up and cut-off values used for the 6mWT distance and the hazard of all-cause mortality according to sex was also not reported.

We reported the 6mWT distance as an absolute value in metres and as a percentage of the predicted value using a published 6mWT reference equation^9^. Reporting absolute and relative 6mWT distance results has additional clinical relevance since physical functional capacity is influenced by factors such as age, sex, and BMI. The use of a specific absolute cut-off value for the absolute distance covered in the 6mWT is problematic. For example, if a 55-year-old man and an 80-year-old woman both walked a distance of 300 m, these results may be interpreted as a major reduction in physical functional capacity for the 55-year-old man and a minor reduction in physical functional capacity for the 80-year-old woman. We chose to compare the patients’ risk of mortality in categories based on distributional quantiles (i.e. tertiles) of the percentage of the predicted 6mWT distance. Our analysis permitted a valid comparison of the patients physical functional capacity with a healthy population with similar demographic and anthropometric characteristics.

While factors such as age, sex, and BMI can influence the 6mWT distance, comorbidities such as diabetes mellitus, renal failure, chronic cerebrovascular disease, and chronic obstructive pulmonary disease can also significantly reduce the 6mWT distance in men and women^28^. Previous studies examining the association between the 6mWT and all-cause mortality have adjusted for only fewer selected comorbidities in their analyses^11,21^. In the present study, we created a comprehensive Elixhauser comorbidity index^27^, based on ICD-10 codes in linked hospital admission data during the previous 12 months that was used as a covariate in the regression models. We found that a longer 6mWT distance was associated with a lower hazard of all-cause mortality and independent of the Elixhauser comorbidity index. The association between a shorter distance walked during the 6mWT and an increased risk of all-cause mortality among cardiac surgery patients was independent of underlying comorbidities.

Previous studies have reported that the distance covered in the 6mWT is determined by the type^21,29^ and complexity of the cardiac surgery procedure and can predict post-hospital discharge mortality^30^. It is important to understand whether the relationship between a shorter 6mWT distance and a higher risk of mortality is confounded or modified by the type and complexity of the cardiac of surgery procedure. We found that patients undergoing combined CABG and valve surgery covered significantly shorter distances during the 6mWT than those patients undergoing isolated CABG or valve only procedures. The direction of the observed association with all-cause mortality was the same in the isolated CABG, valve and combined CABG and valve cardiac surgery patient groups. Combined CABG and valve surgery is a multifaceted intervention addressing simultaneously intricate myocardial revascularisation and valvular repair or replacement. The complexity of addressing coronary and valvular pathologies simultaneously entails a broader physiological impact including greater haemodynamic perturbations, and a more substantial systemic inflammatory response and oxidative stress. Consequently the cumulative effect of addressing coronary and valvular pathologies can contribute to a disenable reduction in postoperative physiological functional capacity, including shorter distances during the 6mWT.

The association between the 6mWT and all-cause mortality however was significant for the isolated CABG and valve patients, but not for patients undergoing combined CABG and valve procedures, potentially due to the smaller sample size of the combined CABG and valve patient group.

Our findings supports the use of the 6mWT for the assessment of patients prior to acute care hospital discharge following cardiac surgery. The 6mWT distance after cardiac surgery can identify patients who may require support to ensure their participation in prescribed phase II cardiac rehabilitation programs following hospital discharge to increase their physical functional capacity^20,28,29^. Supervised exercise programs following cardiac surgery are important determinants of physical functional capacity outcomes, as they can limit the effect of cardiac, geriatric, operative, and postoperative complications^2^. The 6mWT can be used to assess the effectiveness of such cardiac rehabilitation programs for these patients. However, further research to investigate interventions that can optimise gains in the 6mWT after cardiac surgery is warranted.

Our study has the following strengths; (i) we included cardiac surgery patients from a single high-volume cardiac surgery centre, allowing the delivery of a standardised postoperative phase 1 cardiac rehabilitation program on all days of the week. This consistent approach to the rehabilitation service delivery also reduced 6mWT test performance variability compared to multicentre studies and (ii) Linkage of patient data to administrative hospital admission and all-cause mortality records also ensured a long-term follow-up with minimum attrition and allowed us to adjust for a wide range of comorbidities from the hospital admission records. Our study had the following limitations; (i) almost a quarter of the participants in the patient cohort did not perform 6mWT for the following reasons; musculoskeletal and neurological impairments, poor exercise tolerance, discharged prior to assessment, and refusal to complete the 6mWT procedures. Those cardiac surgery patients who did not have 6mWT results compared to cardiac surgery patients who did were more likely to die during follow-up (15% vs. 36%; Supplementary Material, Table S3); (ii) there were fewer female patients in our patient cohort, however the male-to-female ratio was similar to that observed in the Australia cardiac surgery population^31^; (iii) we could not adjust for left ventricular ejection fraction which is a strong predictor of mortality among cardiac surgery patients^21^; (iv) we did not have data on SpO_2_ decline and unintended stops during the 6mWT, which could provide insights into the relationship between respiratory function and exercise capacity; and (v) patients completed one 6MWT at the time of hospital discharge along a corridor length of 43.5 m instead of the recommended 30 m due to the physiotherapists practical time constraints. Although patients who had not undergone a 6MWT at the time of admission did not undertake a 'practice walk', it is important to note that patients engaged in continuous walking as the primary mode of exercise.

## Conclusion

A greater distance walked during the 6mWT immediately before hospital discharge was strongly and independently associated with a lower hazard of all-cause mortality in a contemporary cohort of cardiac surgery patients. The 6mWT should be considered in the postoperative assessment and discharge planning for patients who undergo cardiac surgery.

### Supplementary Information

Supplementary Information 1.

Supplementary Information 2.

## Data Availability

All relevant data are within the manuscript and its Supplementary Materials files.
