# Supplementary material for: Six-minute walk test distance at time of hospital discharge is strongly and independently associated with all-cause mortality following cardiac surgery
Source: Sci Rep. 2024 Jan 30;14:2493. doi: 10.1038/s41598-024-52601-7 (PMC10827724; doi:10.1038/s41598-024-52601-7)
Supplement: Supplementary file 2 — Supplementary Information 2. [file 41598_2024_52601_MOESM2_ESM.docx]

**Six-minute walk test distance at time of hospital discharge is strongly and independently associated with all-cause mortality following cardiac surgery**

**Supplementary materials**

**
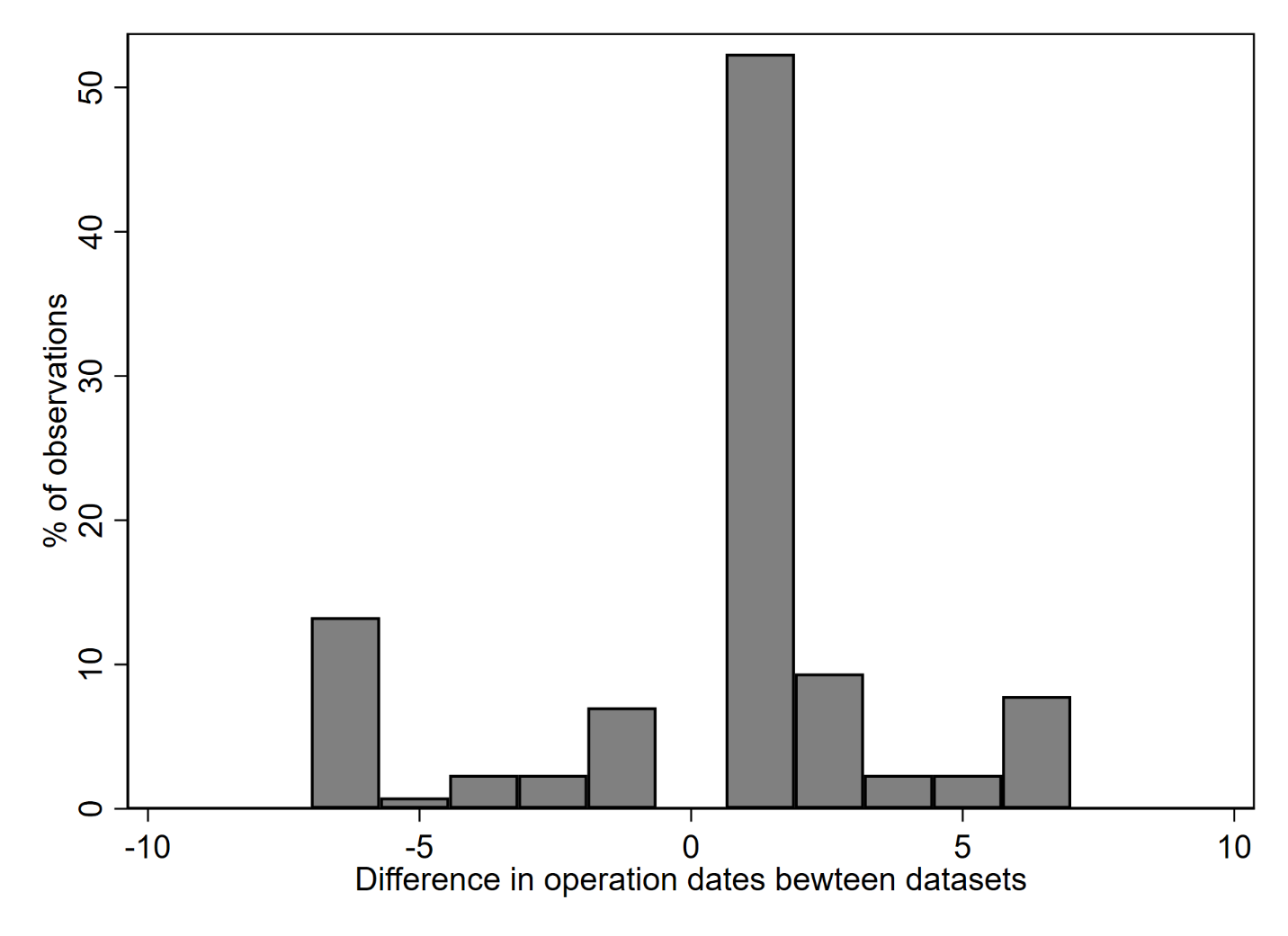
**

**Figure S1: Distribution of differences in operation dates between CuPID dataset and APDC dataset.**

The operation dates in the CuPID and APDC datasets were exactly matched for 89% of the cohort. Among the patients without an exact date match, more than half had a discrepancy of ±1 day between the records

| **Table S1: Comparison of baseline characteristics between those who had 6mWT and who did not have 6mWT information** | | | | |
| --- | --- | --- | --- | --- |
|  |  |  |  |  |
| **Variables** | **Overall** | **Did not have 6mWT** | **Had 6mWT** | **P-value** |
| No. of patients (% of total) | 1599 (100.0) | 406 (25.4) | 1193 (74.6) |  |
| Age (years), mean (SD) | 64.8 (15.8) | 71.6 (15.2) | 62.5 (15.3) | <0.001 |
| Female, n (%) | 482 (30.1) | 162 (39.9) | 320 (26.8) | <0.001 |
| Height (m), mean (SD) | 1.7 (0.1) | 1.7 (0.1) | 1.7 (0.1) | <0.001 |
| Weight (kg), mean (SD) | 82.0 (18.2) | 79.9 (20.3) | 82.7 (17.4) | 0.008 |
| BMI (kg/m2), mean (SD) | 28.6 (5.4) | 28.7 (5.9) | 28.6 (5.2) | 0.695 |
| Current or past smoker, n (%) | 632 (39.5) | 165 (40.6) | 467 (39.1) | 0.594 |
| Operative variables |  |  |  |  |
| Operation performed, n (%) |  |  |  |  |
| CABG | 740 (46.3) | 136 (33.5) | 604 (50.6) | <0.001 |
| CABG + valve | 186 (11.6) | 75 (18.5) | 111 (9.3) |  |
| Valve | 519 (32.5) | 133 (32.8) | 386 (32.4) |  |
| Others | 118 (7.4) | 26 (6.4) | 92 (7.7) |  |
| Urgent procedure, n (%) | 437 (27.3) | 131 (32.3) | 306 (25.6) | <0.001 |
| Reoperative procedure, n (%) | 158 (9.9) | 39 (9.6) | 119 (10.0) | 0.752 |
| Operation time in minutes, mean (SD) | 215.8 (76.8) | 223.2 (91.1) | 213.4 (71.6) | 0.031 |
| Perfusion time in minutes, mean (SD) | 84.4 (40.5) | 91.6 (46.3) | 82.2 (38.3) | <0.001 |
| Total ventilation time in hours, mean (SD) | 14.8 (39.7) | 30.4 (75.6) | 9.9 (14.1) | <0.001 |
| Postoperative variables |  |  |  |  |
| Post-operative length of stay in days, mean (SD) | 9.5 (7.4) | 13.2 (13.4) | 8.4 (3.2) | <0.001 |
| Postoperative complications, n (%) | 465 (29.1) | 193 (47.5) | 272 (22.8) | <0.001 |
| Home discharge, n (%) | 1108 (69.3) | 123 (30.3) | 985 (82.6) | <0.001 |

| Table S2: Selected baseline characteristics, by % of theoretical predicted value | | | |  |
| --- | --- | --- | --- | --- |
| Variables | **Tertile 1** | **Tertile 2** | **Tertile 3** | **p-value** |
| No. of patients (% of total) | 376 (33.4) | 376 (33.4) | 375 (33.3) |  |
| Age in years, mean (SD) | 60.9 (16.4) | 64.1 (12.0) | 67.6 (8.9) | <0.001 |
| Female, n (%) | 125 (33.2) | 82 (21.8) | 78 (20.8) | <0.001 |
| Height in meter, mean (SD) | 1.7 (0.1) | 1.7 (0.1) | 1.7 (0.1) | 0.05 |
| Weight in kg, mean (SD) | 80.7 (18.9) | 85.0 (17.3) | 84.3 (14.9) | 0.002 |
| BMI in kg/m2, mean (SD) | 28.4 (5.6) | 29.1 (5.1) | 28.8 (4.5) | 0.155 |
| Current or past smoker, n (%) | 152 (40.4) | 149 (39.6) | 158 (42.1) | 0.047 |
| Slow vital capacity in Litres, mean (SD) | 1.9 (0.7) | 2.2 (0.7) | 2.4 (0.6) | <0.001 |
| SpO2 level at rest, mean (SD) | 96.0 (5.7) | 96.2 (2.6) | 96.0 (5.5) | 0.347 |
| Systolic blood pressure (in mmHg) at rest, mean (SD) | 125.9 (18.7) | 124.7 (19.2) | 126.4 (17.6) | 0.005 |
| Diastolic blood pressure (in mmHg) at rest, mean (SD) | 69.0 (10.2) | 70.8 (10.4) | 71.5 (9.7) | 0.024 |
| Mean arterial pressure (in mmHg) at rest, mean (SD) | 89.2 (13.2) | 90.0 (12.5) | 91.6 (12.7) | 0.05 |
| Comorbidities |  |  |  |  |
| Previous cardiac surgery, n (%) | 143 (38.0) | 106 (28.2) | 98 (26.1) | 0.001 |
| Elixhauser comorbidities, n (%) |  |  |  | <0.001 |
| 0-1 | 87 (23.1) | 115 (30.6) | 112 (29.9) |  |
| 2-4 | 238 (63.3) | 229 (60.9) | 244 (65.1) |  |
| 5 or more | 51 (13.6) | 32 (8.5) | 19 (5.1) |  |
| Operative variables |  |  |  |  |
| Operation performed, n (%) |  |  |  | <0.001 |
| CABG | 176 (46.8) | 202 (53.7) | 223 (59.5) |  |
| CABG + valve | 44 (11.7) | 31 (8.2) | 34 (9.1) |  |
| Valve | 137 (36.4) | 124 (33.0) | 109 (29.1) |  |
| Others | 19 (5.1) | 19 (5.1) | 9 (2.4) |  |
| Urgent procedure, n (%) | 105 (27.9) | 109 (29.0) | 87 (23.2) | <0.001 |
| Reoperative procedure, n (%) | 54 (14.4) | 30 (8.0) | 20 (5.3) | <0.001 |
| Operation time in minutes, mean (SD) | 231.2 (77.5) | 213.2 (72.0) | 201.3 (60.7) | <0.001 |
| Perfusion time in minutes, mean (SD) | 90.4 (39.6) | 82.2 (40.5) | 76.7 (31.9) | <0.001 |
| Total ventilation time in hours, mean (SD) | 10.9 (12.8) | 9.7 (11.9) | 9.8 (17.9) | 0.447 |
| Postoperative variables |  |  |  |  |
| Postoperative length of stay in days, mean (SD) | 8.9 (3.7) | 8.5 (3.1) | 7.9 (2.7) | 0.001 |
| Postoperative complications, n (%) | 92 (24.5) | 95 (25.3) | 79 (21.1) | 0.355 |
| Home discharge, n (%) | 287 (76.3) | 307 (81.6) | 328 (87.5) | <0.001 |

**
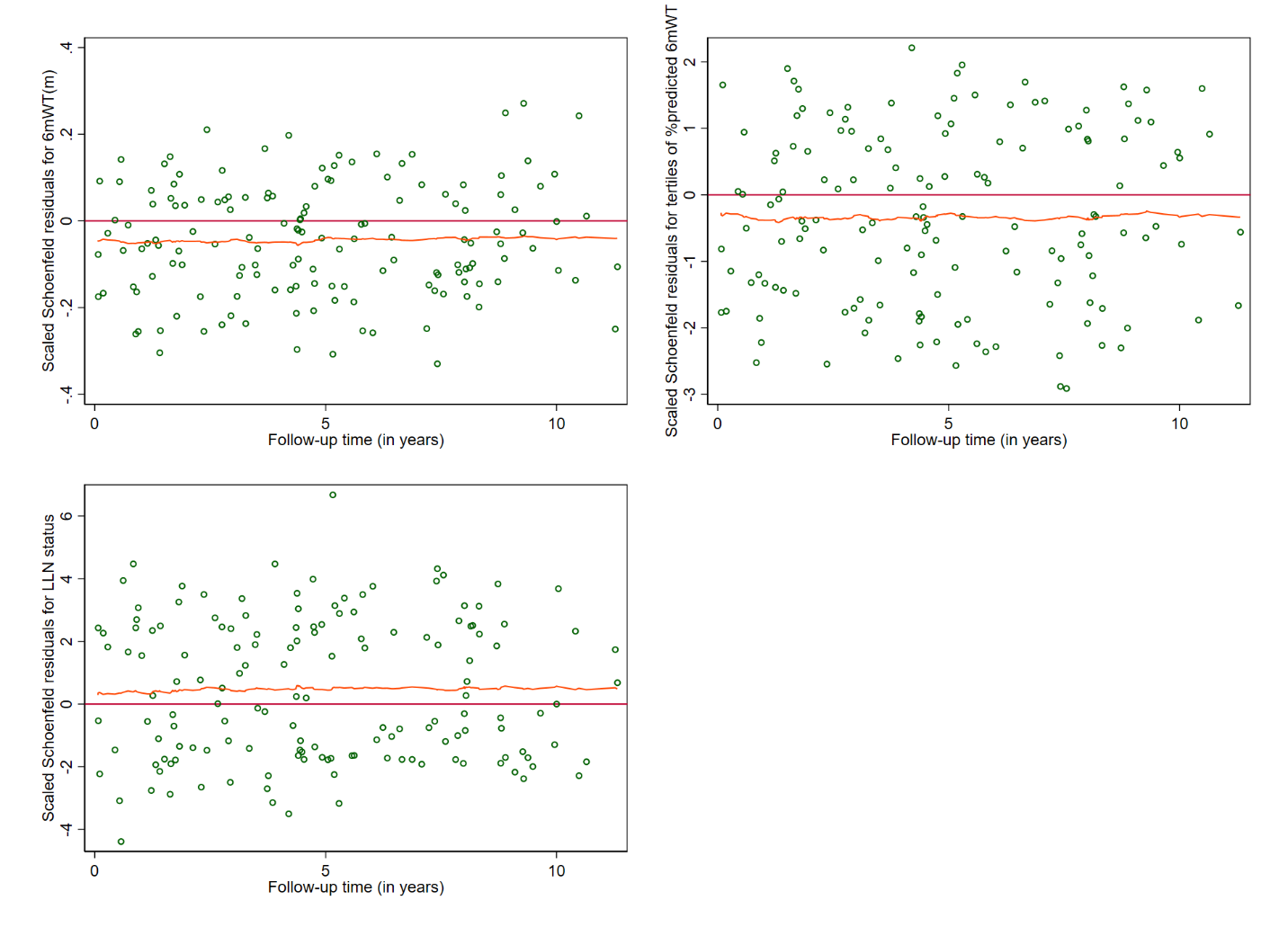
**

**Figure S2: Testing the proportionality assumption by plotting the scaled Schoenfeld residuals.**

A horizontal line in the graphs indicates that there is no violation of the proportionality assumption. The tests for the proportionality of the model were also not significant.

| **Table S3: Mortality among those who did and did not complete the six-minute walk test** | | |
| --- | --- | --- |
|  | **Survived during follow-up** | **Died during follow-up** |
| Completed | 1048 (84.9) | 186 (15.1) |
| Did not complete | 275 (63.8) | 156 (36.2) |
|  |  |  |
| **Reason for not completing** |  |  |
| Discharged prior to assessment | 36 (85.7) | 6 (14.3) |
| Non-English-speaking background | 16 (100.0) | 0 (0.0) |
| Musculoskeletal/neurological/peripheral vascular impairments precluding unaided mobility | 59 (52.2) | 54 (47.8) |
| Other causes | 95 (65.5) | 50 (34.5) |
| Other cardiac reasons | 12 (100.0) | 0 (0.0) |
| Refused | 22 (73.3) | 8 (26.7) |
| Poor exercise tolerance | 31 (64.6) | 17 (35.4) |
| In-hospital death | 0 (0.0) | 17 (100.0) |
